# Supplementary material for: In Silico Prediction of Tetrastatin-Derived Peptide Interactions with αvβ3 and α5β1 Integrins
Source: Pharmaceuticals (Basel). 2025 Jun 21;18(7):940. doi: 10.3390/ph18070940 (PMC12298672; doi:10.3390/ph18070940)
Supplement: Supplementary file 1 [file pharmaceuticals-18-00940-s001.zip › pharmaceuticals-3671864-supplementary.pdf]

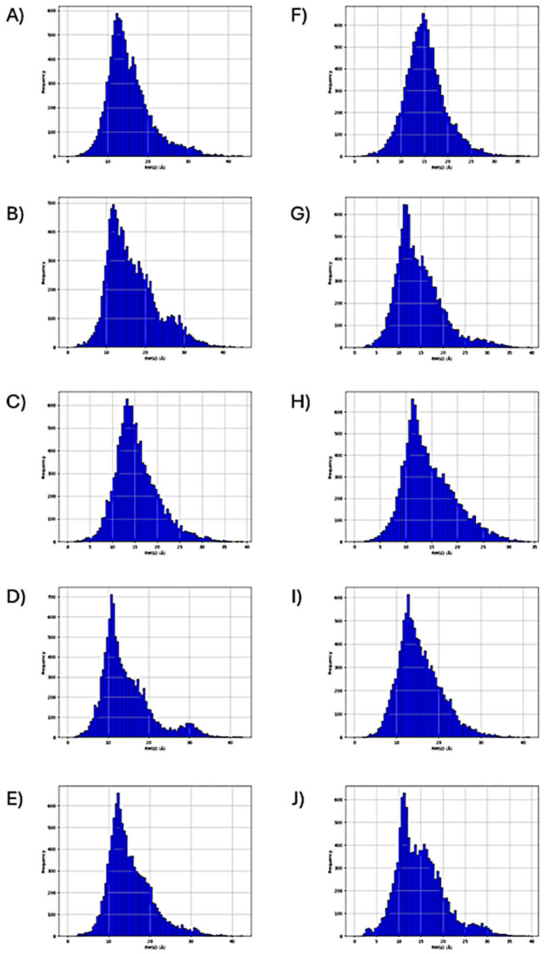

Figure S1a: RMSD analysis of the predominant conformations from clusters of docking poses without disulfide bridges, based on the best pose from the 150 docking results on α5β1 integrin. A-E: Poses from Cluster 1, without a disulfide bridge. F-J: Poses from Cluster 2, also without a disulfide bridge. A. and F. represent QS-13-1, B. and G. represent QS-13-2, C. and H. represent QS-13-3, D. and I. represent QS-13-4, and E. and J. represent QS-13-5. The analysis highlights structural similarities and differences across clusters, with RMSD values expressed in Ångströms and a bin size of 0.5 Å.

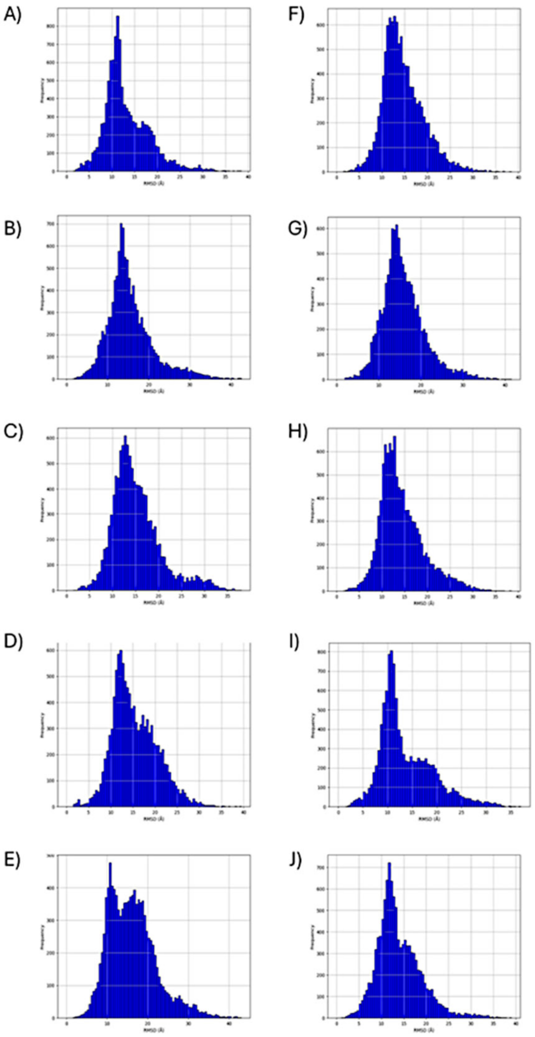

Figure S1b: RMSD analysis of the predominant conformations from clusters of docking poses with disulfide bridges, based on the best pose from the 150 docking results on α5β1 integrin. A-E: Poses from Cluster 1, with a disulfide bridge. F-J: Poses from Cluster 2, also with a disulfide bridge. A. and F. represent QS-13-1, B. and G. represent QS-13-2, C. and H. represent QS-13-3, D. and I. represent QS-13-4, and E. and J. represent QS-13-5. The analysis highlights structural similarities and differences across clusters, with RMSD values expressed in Ångströms and a bin size of 0.5 Å.

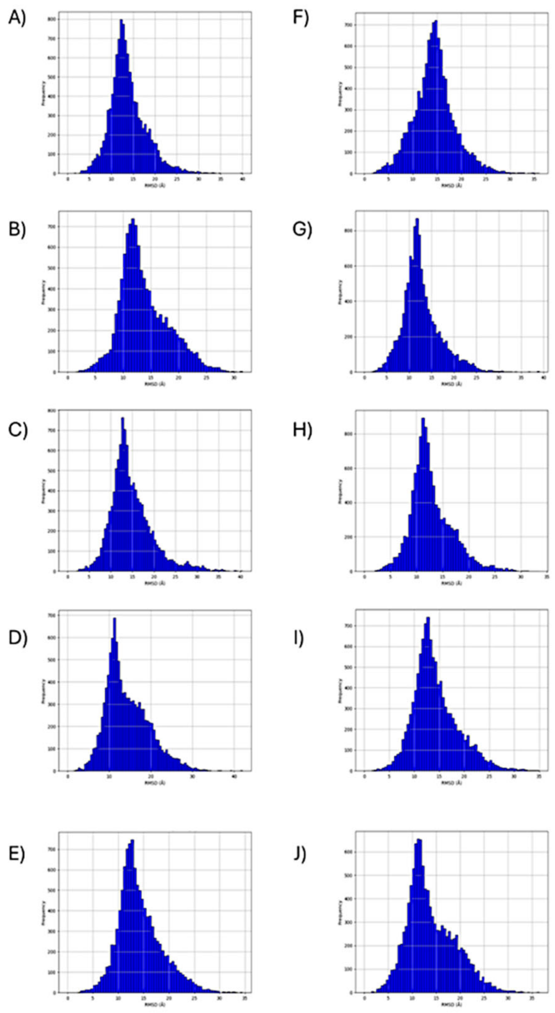

Figure S1c: RMSD analysis of the predominant conformations from clusters of docking poses without disulfide bridges, based on the best pose from the 150 docking results on αVβ3 integrin. A-E: Poses from Cluster 1, without a disulfide bridge. F-J: Poses from Cluster 2, also without a disulfide bridge. A. and F. represent QS-13-1, B. and G. represent QS-13-2, C. and H. represent QS-13-3, D. and I. represent QS-13-4, and E. and J. represent QS-13-5. The analysis highlights structural similarities and differences across clusters, with RMSD values expressed in Ångströms and a bin size of 0.5 Å.

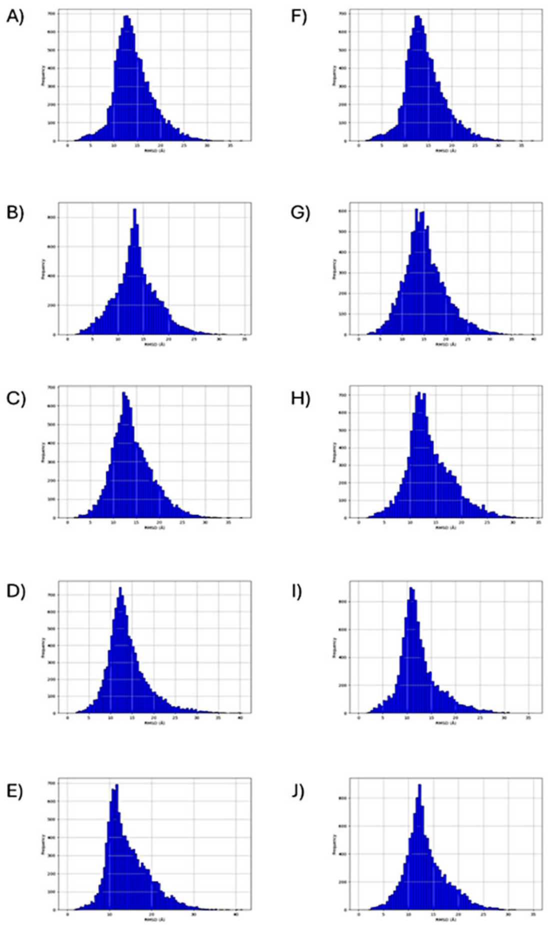

Figure S1d: RMSD analysis of the predominant conformations from clusters of docking poses with disulfide bridges, based on the best pose from the 150 docking results on αVβ3 integrin. A-E: Poses from Cluster 1, with a disulfide bridge. F-J: Poses from Cluster 2, also with a disulfide bridge. A. and F. represent QS-13-1, B. and G. represent QS-13-2, C. and H. represent QS-13-3, D. and I. represent QS-13-4, and E. and J. represent QS-13-5. The analysis highlights structural similarities and differences across clusters, with RMSD values expressed in Ångströms and a bin size of 0.5 Å.

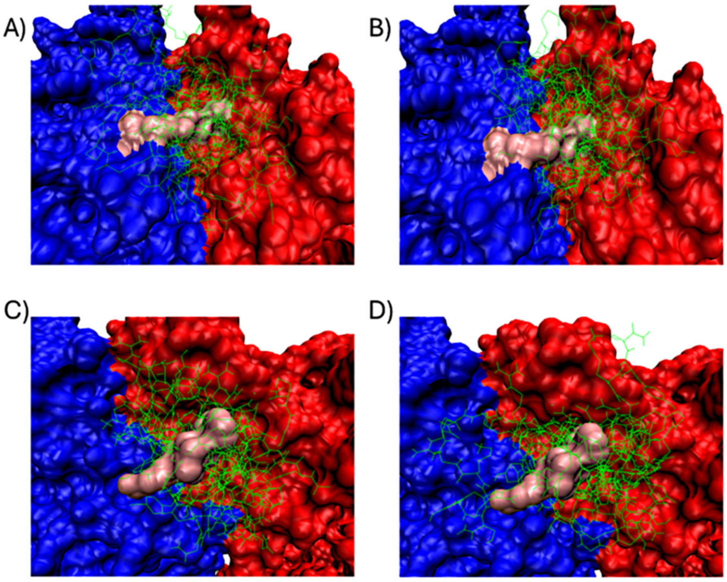

Figure S2: Superposition of the best docking poses from the two predominant clusters of peptides (with and without disulfide bridges) onto the co-crystallized structures of integrins (3VI4 for α5β1 and 1L5G for αVβ3). The α subunit is shown in blue surface, the β subunit in red surface, the RGD peptide in pink surface, and the docked peptide poses in green lines. Visualized using VMD. A and B: α5β1 integrin (3VI3). C and D: αVβ3 integrin (4G1M). A and C represent peptides without disulfide bridges, while B and D represent peptides with disulfide bridges.

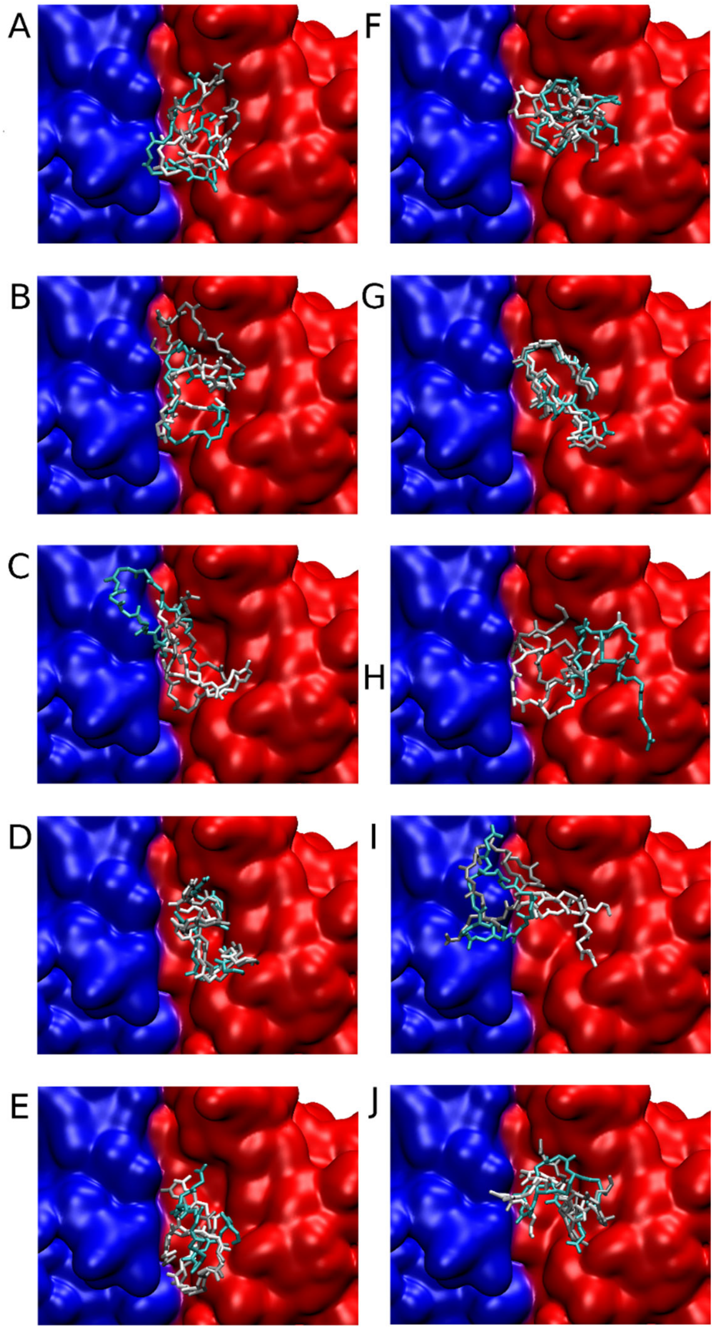

Figure S3: Superimposition of the three predominant conformations of peptides derived from QS-13 docked onto the αv (blue surface) β3 (red surface) integrin. A-E: Peptides without disulfide bridge between cysteine residues; F-J: Peptides with a disulfide bridge. A and F: QS-13-1, B and G: QS-13-2, C and H: QS-13-3, D and I: QS-13-4, E and J: QS-13-5. For clarity, only the backbone of the peptide is shown using a licorice representation. The first, second, and third positions are colored cyan, silver, and white, respectively.

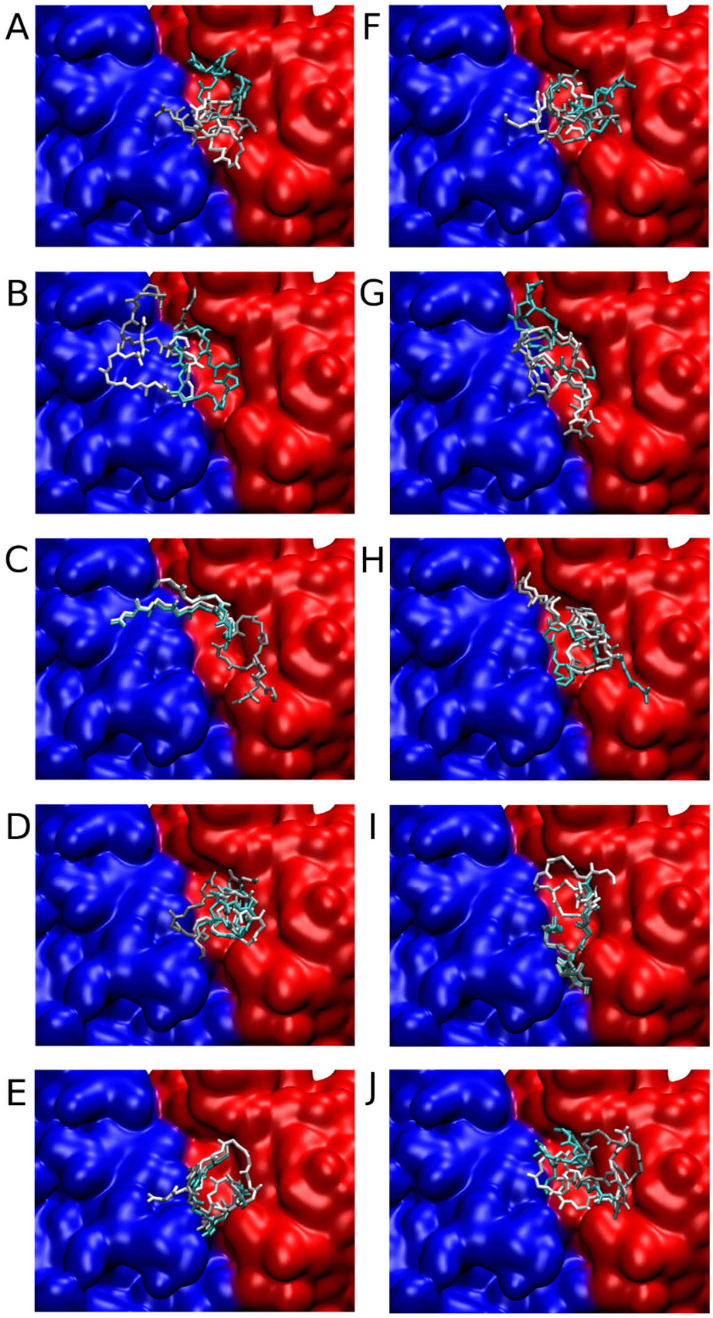

Figure S4: Superimposition of the three predominant conformations of peptides derived from QS-13 docked onto the integrin α5 (blue surface) β1 (red surface). A-E Correspond to peptides without disulfide bridges between cysteine residues, while figures F-J are associated with peptides featuring a disulfide bridge. A and F: QS-13-1, B and G: QS-13-2, C and H: QS-13-3, D and I: QS-13-4, E and J: QS-13-5. For clarity, only the backbone of the peptide is shown using a licorice representation. The first, second, and third positions are colored cyan, silver, and white, respectively.

QS13-1

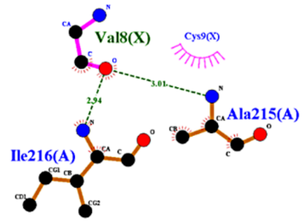

QS13-2

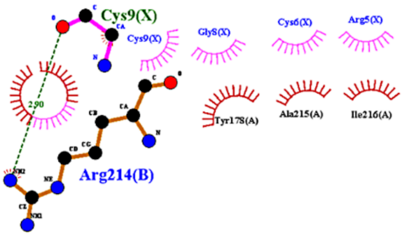

QS13-3

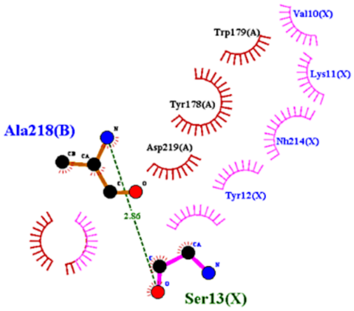

QS13-4

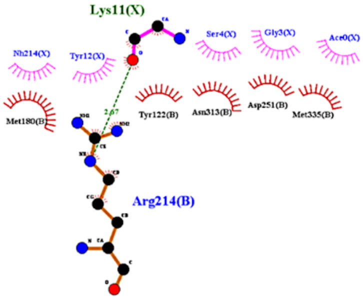

QS13-5

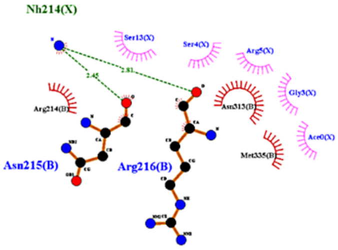

Figure S5a: LigPlot analysis of interactions between QS-13-derived peptides without a disulfide bond (Cluster 1) and αVβ3 integrin. A: Interactions with the α subunit, B: Interactions with the β subunit, X: Interacting residues from the QS-13-derived peptide.

QS13-1

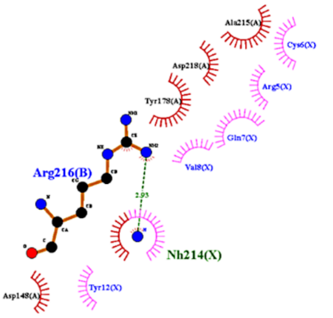

QS13-2

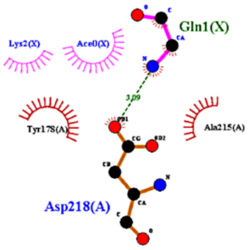

QS13-3

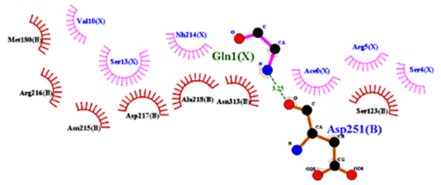

QS13-4

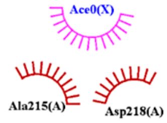

QS13-5

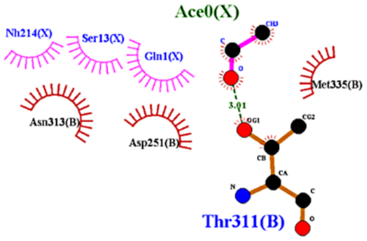

Figure S5b: LigPlot analysis of interactions between QS-13-derived peptides without a disulfide bond (Cluster 2) and αVβ3 integrin. A: Interactions with the α subunit, B: Interactions with the β subunit, X: Interacting residues from the QS-13-derived peptide.

QS13-1

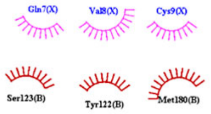

QS13-2

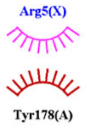

QS13-3

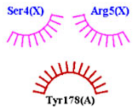

QS13-4

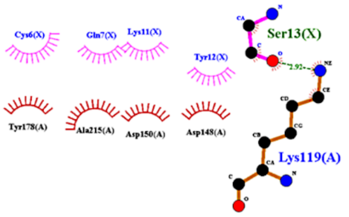

QS13-5

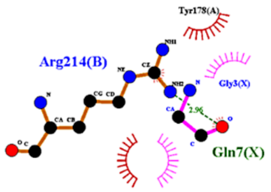

Figure S5c: LigPlot analysis of interactions between QS-13-derived peptides with a disulfide bond (Cluster 1) and αVβ3 integrin. A: Interactions with the α subunit, B: Interactions with the β subunit, X: Interacting residues from the QS-13-derived peptide.

QS13-1

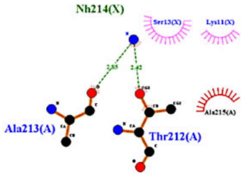

QS13-2

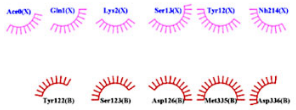

QS13-3

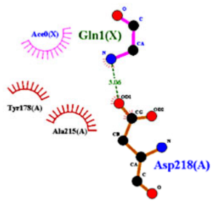

QS13-4

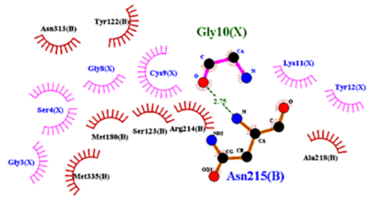

QS13-5

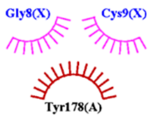

Figure S5d: LigPlot analysis of interactions between QS-13-derived peptides with a disulfide bond (Cluster 2) and αVβ3 integrin. A: Interactions with the α subunit, B: Interactions with the β subunit, X: Interacting residues from the QS-13-derived peptide.

QS13-1

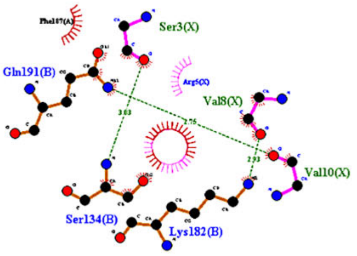

QS13-2

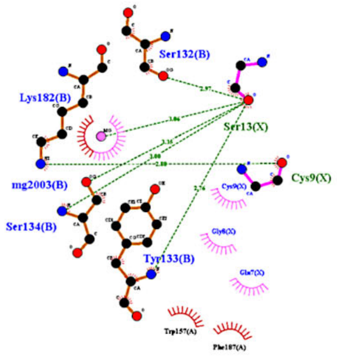

QS13-3

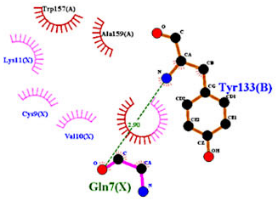

QS13-4

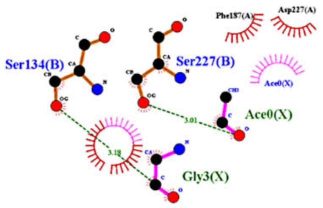

QS13-5

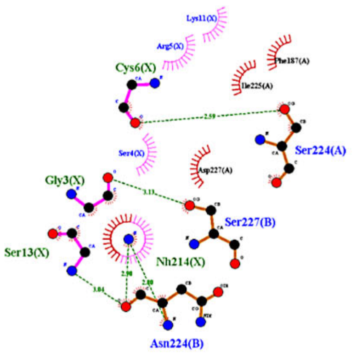

Figure S5e: LigPlot analysis of interactions between QS-13-derived peptides without a disulfide bond (Cluster 1) and α5β1 integrin. A: Interactions with the α subunit, B: Interactions with the β subunit, X: Interacting residues from the QS-13-derived peptide.

QS13-1

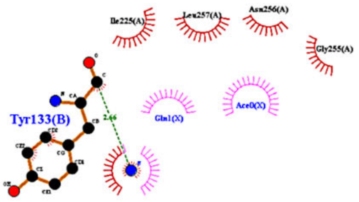

QS13-2

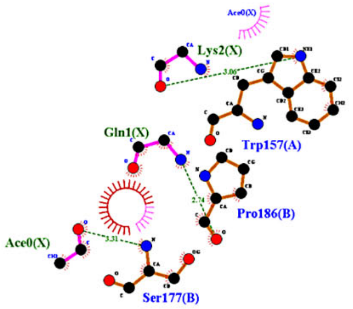

QS13-3

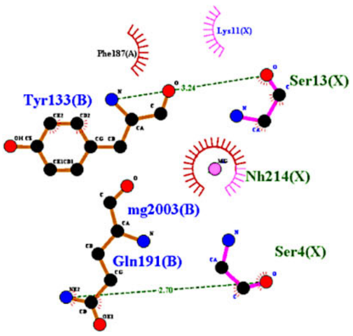

QS13-4

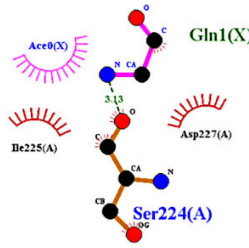

QS13-5

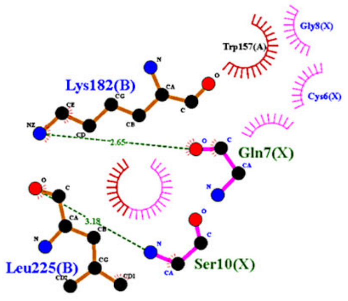

Figure S5f: LigPlot analysis of interactions between QS-13-derived peptides without a disulfide bond (Cluster 2) and α5β1 integrin. A: Interactions with the α subunit, B: Interactions with the β subunit, X: Interacting residues from the QS-13-derived peptide.

QS13-1

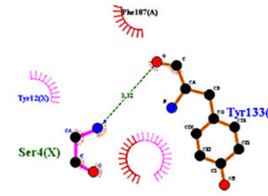

QS13-2

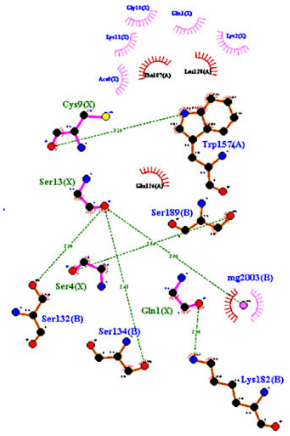

QS13-3

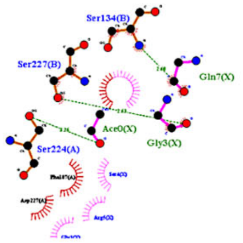

QS13-4

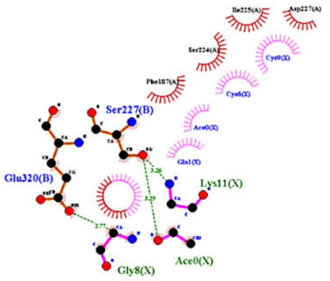

QS13-5

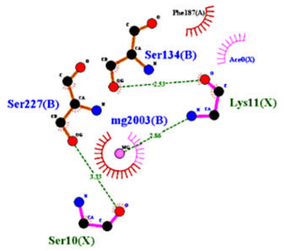

Figure S5g: LigPlot analysis of interactions between QS-13-derived peptides with a disulfide bond (Cluster 1) and α5β1 integrin. A: Interactions with the α subunit, B: Interactions with the β subunit, X: Interacting residues from the QS-13-derived peptide.

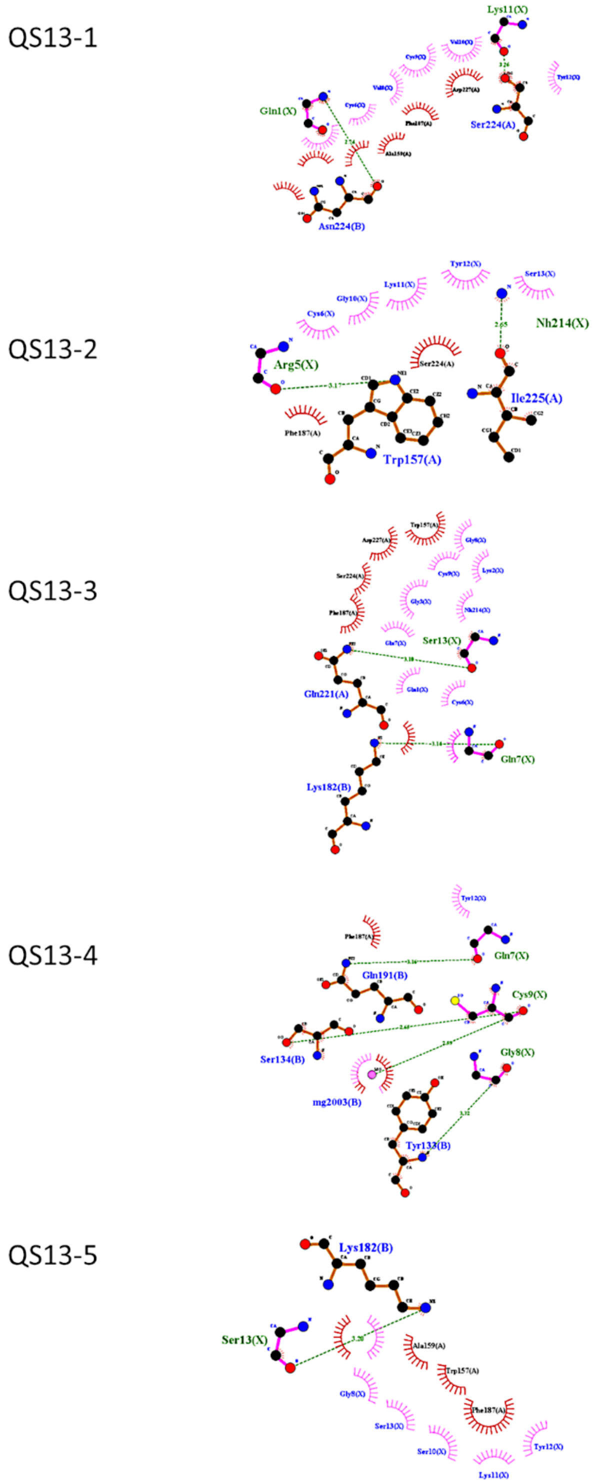

Figure S5h: LigPlot analysis of interactions between QS-13-derived peptides with a disulfide bond (Cluster 2) and α5β1 integrin. A: Interactions with the α subunit, B: Interactions with the β subunit, X: Interacting residues from the QS-13-derived peptide.
